# The Aging Process of Facial Muscles

**DOI:** 10.1111/jocd.70590

**Published:** 2025-12-18

**Authors:** Kyu‐Ho Yi, Jovian Wan

**Affiliations:** ^1^ Division in Anatomy and Developmental Biology, Department of Oral Biology, Human Identification Research Institute, BK21 FOUR Project Yonsei University College of Dentistry Seoul Korea; ^2^ You and I Clinic Seoul Republic of Korea; ^3^ Medical Research Inc. Wonju Korea

## 1 Introduction

Facial aging has traditionally been attributed to passive processes such as skin laxity, fat atrophy, and gravitational descent [1, 2, 3]. However, emerging anatomical and clinical evidence reveals a more dynamic mechanism: progressive muscle contracture actively reshapes facial architecture, leading to cascading structural changes. Cadaveric dissections and imaging studies demonstrate that chronic muscle shortening and increased resting tone fundamentally alter facial form and function, challenging conventional aging paradigms.

This clinical commentary explores the pivotal role of muscular contracture in facial aging, supported by anatomical observations, ethnic variations in muscle architecture, and biomechanical interactions between muscle, fat, and bone. We also discuss clinical implications for aesthetic treatments, emphasizing the need for contracture‐release strategies to achieve natural rejuvenation.

## 2 Periorbital Transformations

**FIGURE 1:**
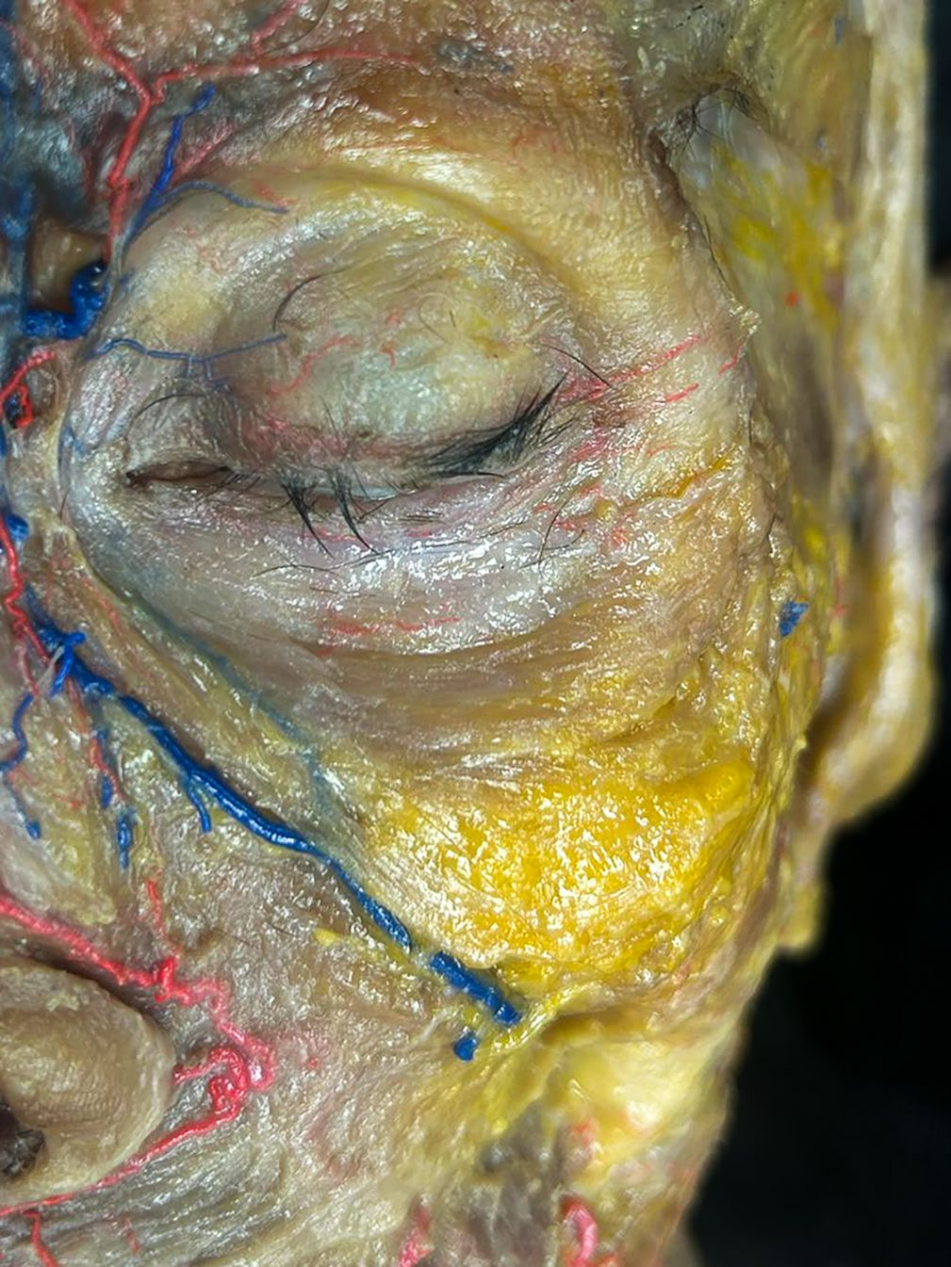
Infraorbital dissection of a 72‐year‐old female donor reveals significant thinning of the superficial fat pad, with near‐complete exposure of the underlying orbicularis oculi muscle. The muscle fibers are visibly prominent through the attenuated subcutaneous layer, demonstrating the anatomical basis for age‐related infraorbital hollowing.

The orbicularis oculi muscle demonstrates profound age‐related alterations. Cadaveric studies show its transition from a thick, concentric band in youth to an attenuated structure in later life. As superficial fat pads diminish, the muscle becomes visible through thinned tissues, contributing to infraorbital hollowing and the appearance of dark circles (Figure 1).

**FIGURE 2:**
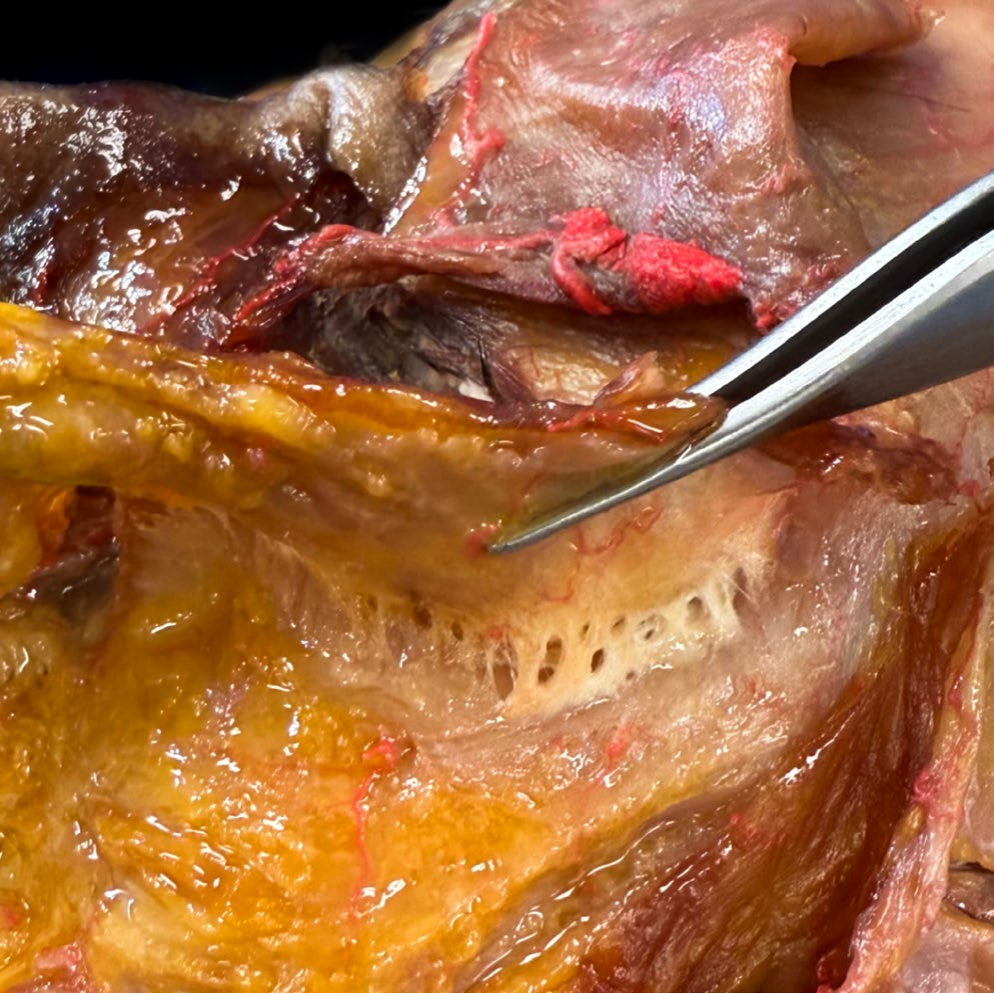
Cadaveric dissection of an elderly donor demonstrates the attenuated orbicularis retaining ligament, showing characteristic age‐related structural weakening. The lax osteocutaneous ligament permits anterior herniation of pre‐septal fat, creating the clinical presentation of malar festoons.

**FIGURE 3:**
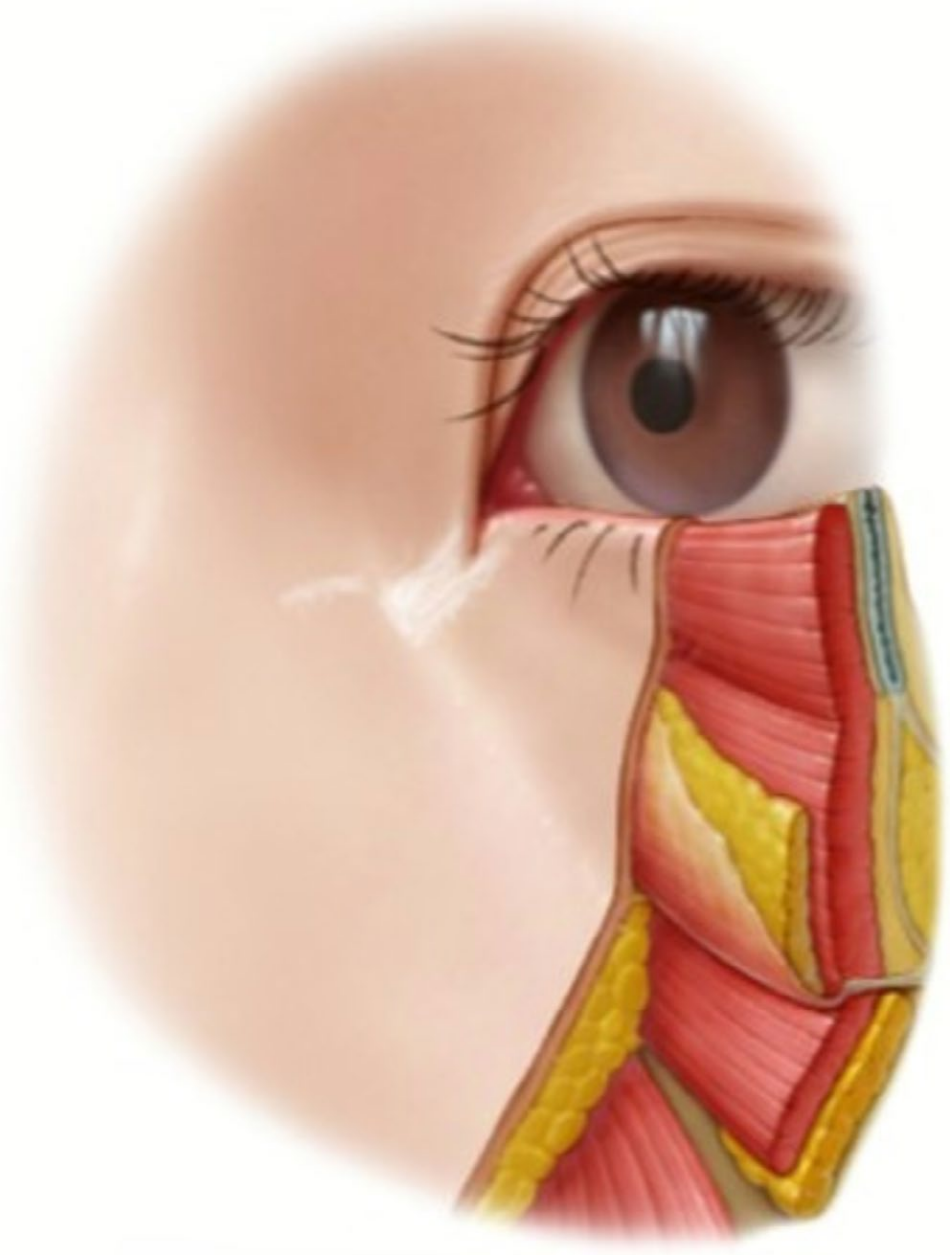
Schematic representation of the tear trough muscle fibers as they course superficially through the subcutaneous fat layer before inserting vertically into the nasojugal dermis. Their unique trajectory explains how age‐related fat atrophy unmasks these fibers, leading to visible depression formation.

The orbicularis retaining ligament, a critical stabilizing structure, weakens with age. This permits pre‐septal fat to bulge above the ligament, forming malar festoons, a phenomenon previously misattributed solely to gravitational descent (Figure 2). Notably, the medial portion of the orbicularis oculi contains vertically oriented fibers that insert directly into the dermis. These “tear trough fibres” become more prominent as overlying fat atrophies, actively contributing to nasojugal groove formation through sustained traction (Figure 3).

## 3 Midface Remodeling

**FIGURE 4:**
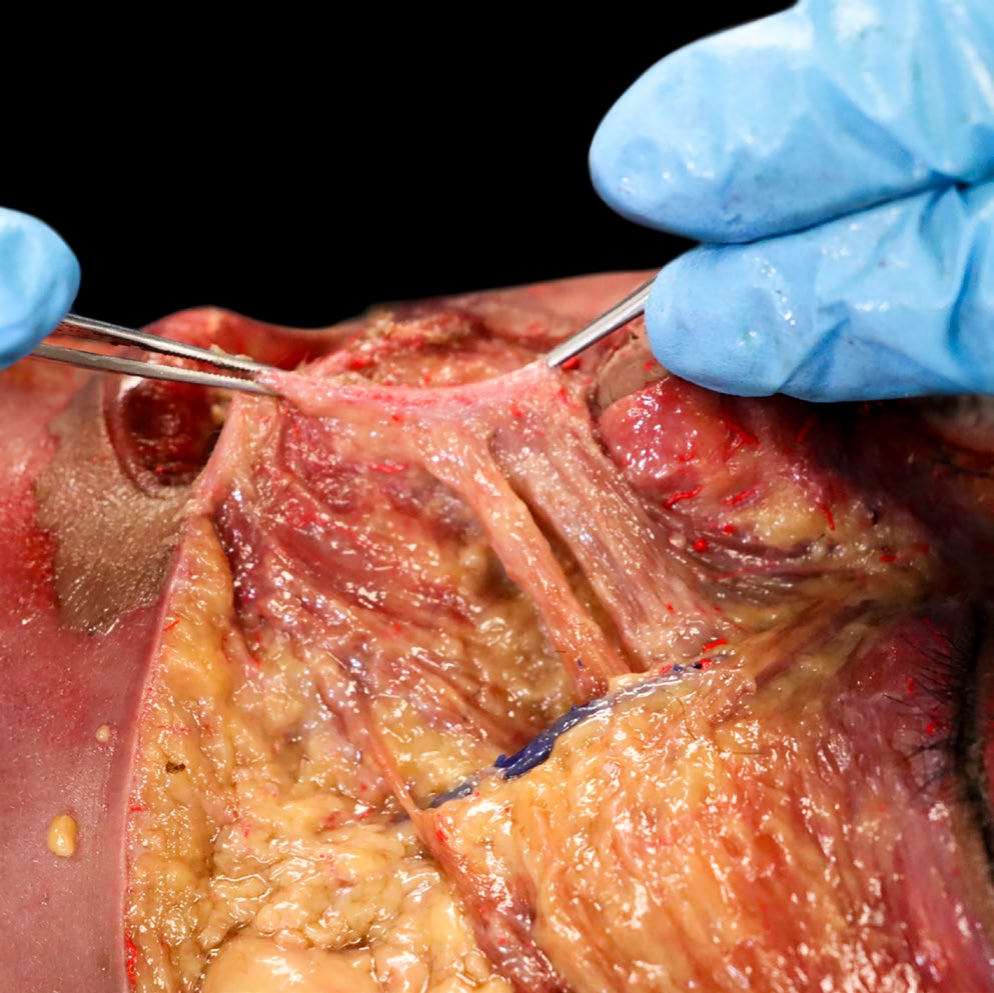
Cadaveric demonstration of lip elevator muscle attachments. This above the nasolabial fold reveals the dermal insertions of the lip elevator muscles along the nasolabial fold region. The Levator Labii Superioris Alaeque Nasi (LLSAN), Levator Labii Superioris (LLS), Zygomaticus major, and Zygomaticus minor muscles all attach to the dermal layer (arrows). Note the convergence of these muscular attachments along the nasolabial fold.

**FIGURE 5:**
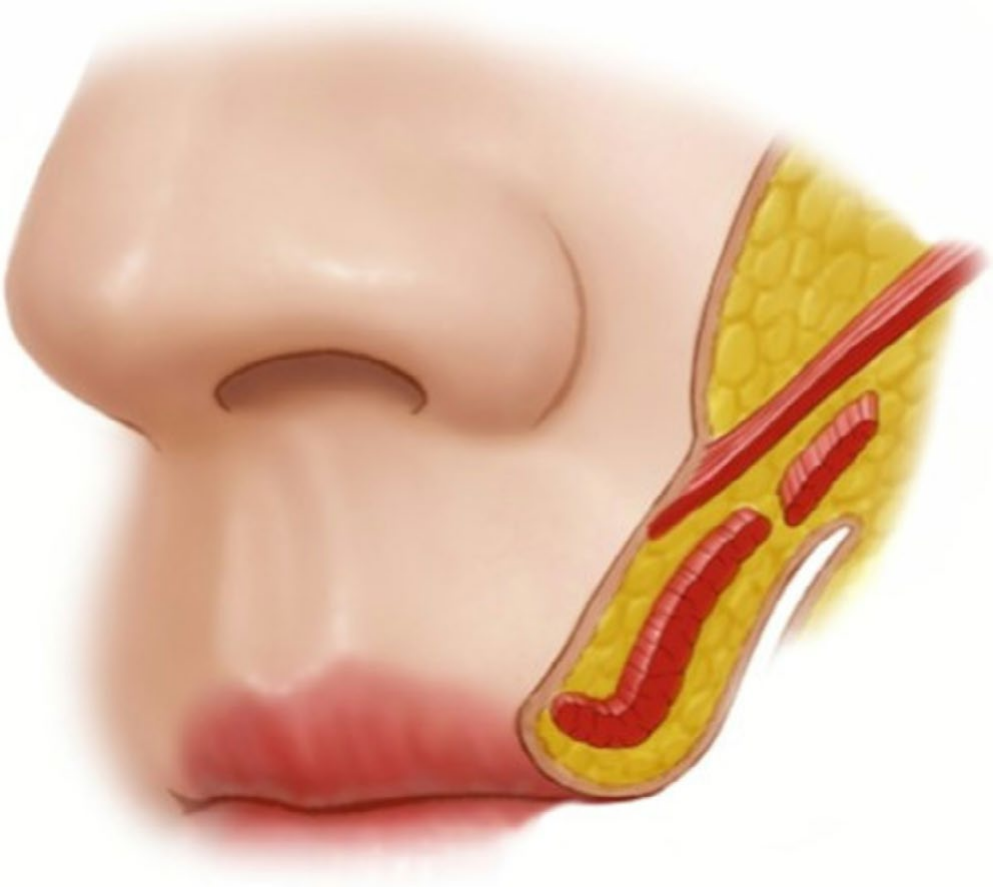
A schematic representation of the zygomaticus major muscle attaching to the dermal layer.

The zygomaticus major exhibits differential aging patterns in its bilayered structure. While superficial fibers attaching to the dermis develop increased rigidity, deeper fibers maintaining connections with the orbicularis oris retain greater elasticity (Figures 4, 5). This architectural divergence explains the clinical phenomenon of preserved dynamic smile capacity coexisting with prominent nasolabial folds. Contracture of superficial fibers actively displaces the nasolabial fat pad superiorly, demonstrating that fold formation results from muscular traction rather than passive volume loss alone.

## 4 Labial Aging Dynamics

**FIGURE 6:**
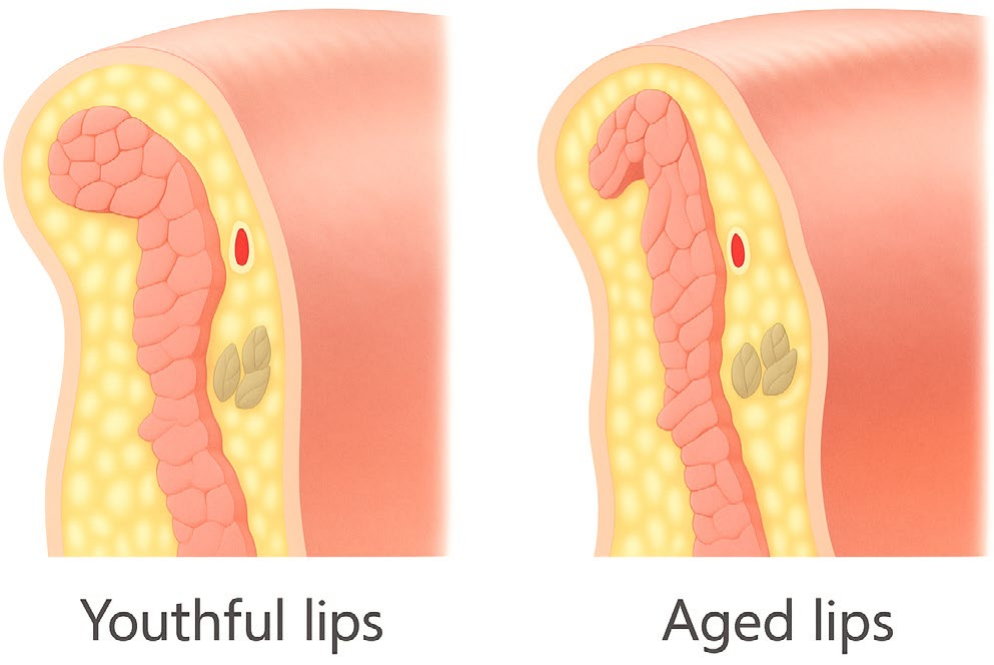
Schematic comparison of youthful versus aged lips demonstrates characteristic fat atrophy and orbicularis oris muscle flattening leading to lip inversion.

The orbicularis oris demonstrates two concurrent aging processes that collectively transform lip architecture. Peripheral fibers develop progressive contracture and elevated resting tone, creating radial compression forces, while marginal fibers maintain structural integrity but lose elasticity. These changes, combined with significant atrophy of superficial labial fat compartments, convert the youthful everted lip profile to an inverted, flattened morphology (Figure 6). Secondary contributions from hypertonic depressor anguli oris further accentuate these aging changes, necessitating comprehensive treatment approaches.

## 5 Lower Face and Cervical Muscular Aging

**FIGURE 7:**
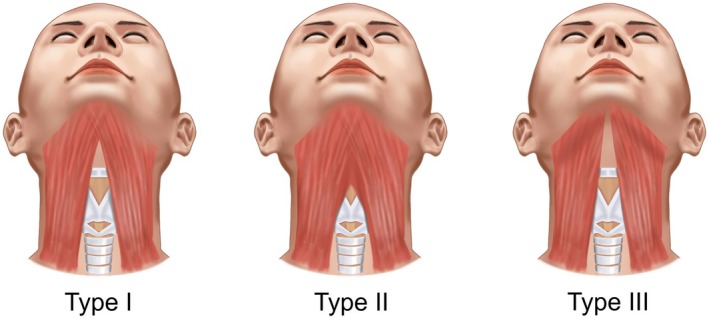
Platysmal decussation patterns show reduced “gobbler neck” appearance in overlapping (Asian‐prevalent) versus non‐overlapping (Caucasian‐prevalent) configurations.

**FIGURE 8:**
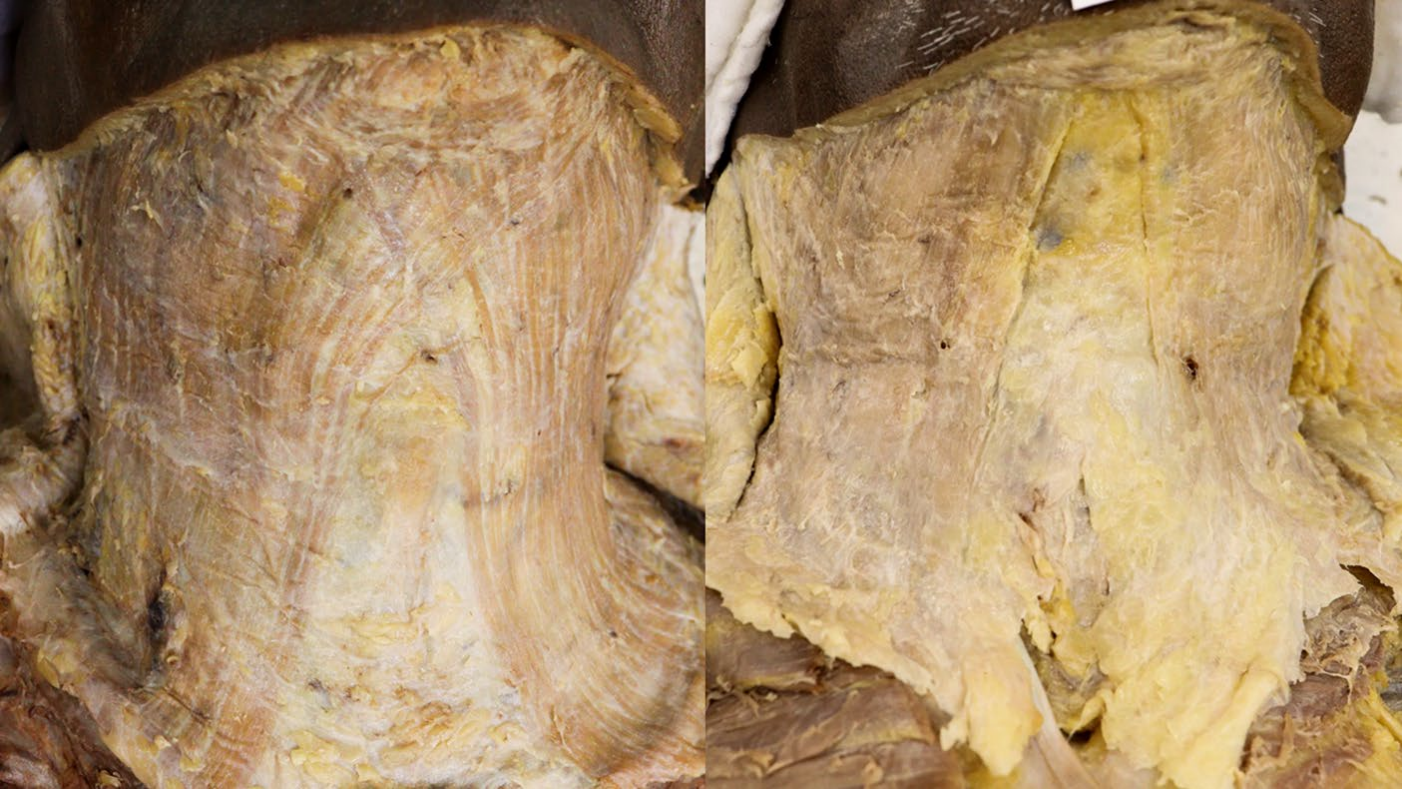
Cadaveric dissection demonstrating platysmal muscle decussation (overlapping) versus non‐decussation (parallel) patterns.

**FIGURE 9:**
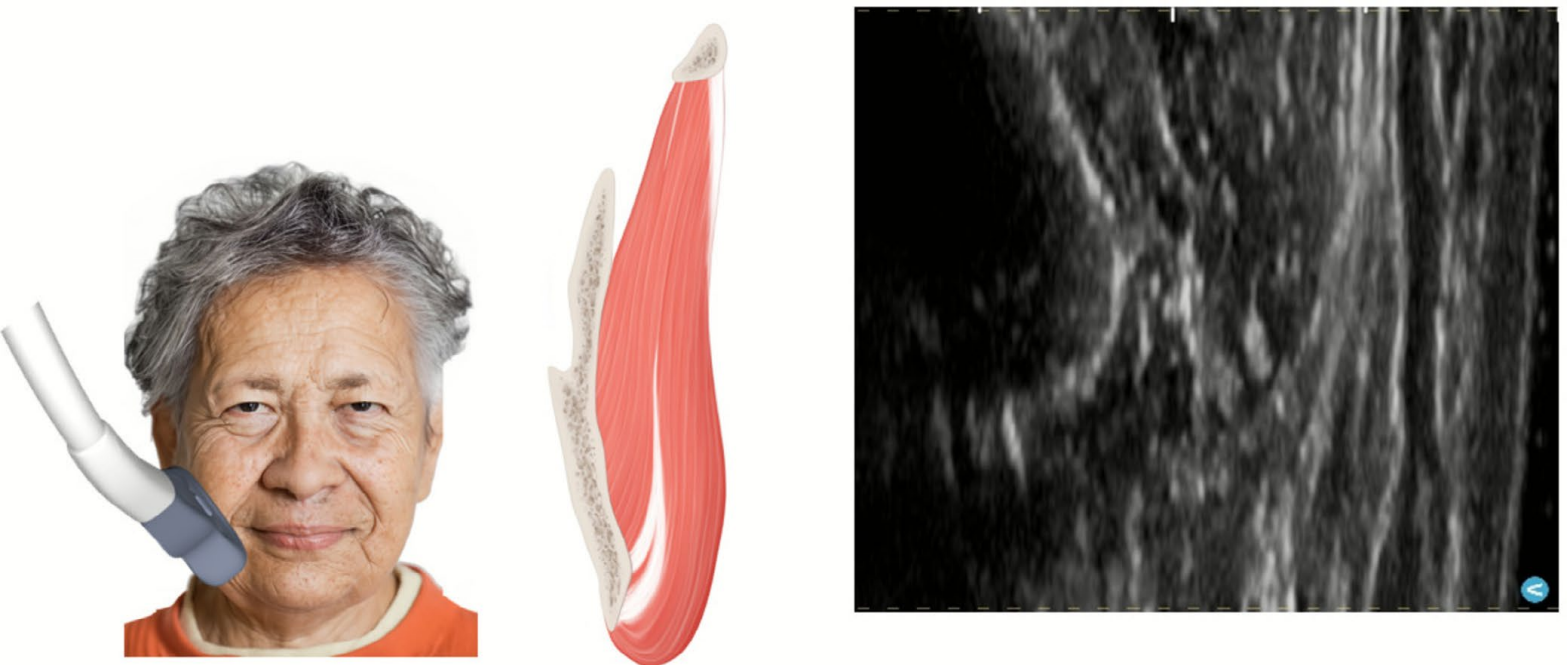
Ultrasound imaging reveals inferior displacement of the masseter muscle beyond the mandibular border in an elderly patient, demonstrating loss of gonial angle definition. The sagging muscle fibers and progressive thinning contribute to submandibular contour changes, challenging the traditional attribution to fat redistribution alone.

The platysma undergoes fibrotic transformation with age, developing increased cross‐linking between collagen fibers while losing elastic support from surrounding tissues. These changes create characteristic banding patterns that vary ethnically, broad anterior bands in patients with decussating fiber patterns versus discrete vertical cords in those with parallel fiber architecture (Figures 7, 8) [4]. Concurrently, deeper masticatory muscles undergo progressive sarcopenia, with the temporalis and masseter exhibiting significant age‐related volume reduction, particularly evident in advanced decades (Figure 9).

## 6 Discussion

The biomechanical relationship between facial muscles and underlying bone structure follows Wolff's law, which dictates that bone remodels in response to mechanical stress. This principle, well established in orthopedics and rehabilitation medicine, explains why weakened facial musculature accelerates bony resorption in aging. When muscles lose tone and contractile strength, they fail to generate sufficient tensile forces on their osseous attachments, creating a biochemical environment favoring osteoclastic activity over bone formation [5, 6, 7, 8].

This phenomenon manifests clinically as characteristic age‐related changes including progressive resorption of the mandibular angle, anterior maxilla, and orbital rims. The masseter and temporalis muscles provide a compelling example; their declining strength with age reduces loading on the mandibular ramus and zygomatic arches, leading to measurable decreases in bone density at these sites. Similarly, diminished tone in the mimetic muscles contributes to resorption along their origins at the piriform aperture and inferior orbital rim. The rehabilitation principle that mechanically loaded bone maintains its strength applies directly to facial aesthetics, explaining why therapeutic approaches should aim to restore physiological muscular tension [8].

Emerging evidence suggests energy‐based modalities may counteract this resorption by stimulating osteoblast activity. Multiwavelength laser therapy shows particular promise through photobiomodulation effects that mimic the beneficial impacts of muscular loading on bone metabolism [9]. This understanding fundamentally alters our approach to facial rejuvenation, as current strategies replacing atrophied fat with denser hyaluronic acid fillers often exacerbate the problem. Aged muscles frequently cannot adequately animate these heavier materials, further reducing mechanical stimulation of underlying bone while creating the unnatural “overfilled” appearance during expression [10, 11].

Future therapeutic directions should prioritize preserving native fat compartments where possible and selecting fillers with density matching residual muscular capacity. Incorporating modalities that directly stimulate osteogenesis while developing muscle‐strengthening protocols for facial rehabilitation could help restore the natural biomechanical balance. By addressing both the muscular and osseous components of aging through this integrated understanding of Wolff's law, we can advance beyond superficial correction toward truly physiological rejuvenation that maintains the face's natural dynamics and expression.

## 7 Conclusion

Facial muscles undergo distinctive aging processes that actively shape morphological changes through architectural remodeling and altered biomechanics. This muscle‐centric paradigm provides a more comprehensive understanding of facial aging, emphasizing the importance of addressing dynamic muscular changes as the foundation for effective rejuvenation. By developing interventions that target these underlying mechanisms, clinicians can achieve more natural, durable outcomes that preserve facial expressivity while reversing the stigmata of aging.

## Author Contributions

All authors have reviewed and approved the article for submission. Conceptualization, Kyu‐Ho Yi, Jovian Wan. Writing – Original Draft Preparation, Kyu‐Ho Yi, Jovian Wan. Writing – Review and Editing, Jovian Wan. Visualization, Kyu‐Ho Yi, Jovian Wan. Supervision, Kyu‐Ho Yi.

## Funding

The authors have nothing to report.

## Ethics Statement

This study did not involve experimental research on human participants or animals. All patients provided written informed consent for the use of their clinical images and relevant information for publication purposes. The procedures described were performed in accordance with the principles of the Declaration of Helsinki.

## Consent

Informed consent was obtained from all participants, with full disclosure of the study's purpose, risks, and confidentiality.

## Conflicts of Interest

The authors declare no conflicts of interest.

## Data Availability

The data that support the findings of this study are available from the corresponding author upon reasonable request.
